# The role of predictability and structure in word stress processing: an ERP study on Cairene Arabic and a cross-linguistic comparison

**DOI:** 10.3389/fpsyg.2014.01151

**Published:** 2014-10-21

**Authors:** Ulrike Domahs, Johannes A. Knaus, Heba El Shanawany, Richard Wiese

**Affiliations:** ^1^Institut für Deutsche Sprache und Literatur, University of CologneCologne, Germany; ^2^Department of Linguistics, Languages and Cultures, University of CalgaryCalgary, AB, Canada; ^3^Department of German, Menoufiya UniversityMenoufiya, Egypt; ^4^Institut für Germanistische Sprachwissenschaft, University of MarburgMarburg, Germany

**Keywords:** metrical structure, word stress perception, Cairene Arabic, Turkish, German, P300 effect, predictability

## Abstract

This article presents neurolinguistic data on word stress perception in Cairene Arabic, in comparison to previous results on German and Turkish. The main goal is to investigate how central properties of stress systems such as predictability of stress and metrical structure are reflected in the prosodic processing of words. Cairene Arabic is a language with a regular foot-based word stress system, leading to highly predictable placement of word stress. An ERP study on Cairene Arabic is reported, in which a stress violation paradigm is used to investigate the factors predictability of stress and foot structure. The results of the experiment show that for Cairene Arabic the internal structure of prosodic words in terms of feet determines prosodic processing. This structure effect is complemented by a frequency effect for stress patterns.

## Introduction

Recent crosslinguistic studies on word stress perception revealed a correlation between the predictability of stress positions in a native language and the sensitivity to stress properties in second languages. In a series of studies utilizing a stress sequence recall paradigm, Dupoux, Peperkamp and colleagues found that speakers of a language with predictable word stress have difficulties to store stress information in abstract phonological representations when learning an L2 with lexical stress (e.g., Dupoux et al., 1997, 2001, 2008, 2010; Peperkamp and Dupoux, 2002; Peperkamp et al., 2010). Within a continuum of predictability ranging from predictable without exceptions to non-predictable, grades of stress-“deafness” were identified as a function of the number of exceptions from a predictable stress position. Speakers of a language with invariable stress (e.g., French) are less sensitive to stress information than speakers of a language with variable stress (e.g., Spanish). Furthermore, the more variable stress positions in a language are the more likely it is that stress information has to be lexically specified. In more recent studies, Peperkamp et al. (2010) suggest the crucial factor for stress sensitivity to be the amount of exceptional stress in a given language. The fewer cases of exceptional stress the more likely that speakers show reduced sensitivity to stress information.

So far, investigations of language specific stress representations have mainly addressed the influence of fixed vs. variable stress. The question arises what kind of stress representation has to be assumed for languages with variable stress that are said to be predictable by means of metrical structure, i.e., by predictable parsing routines of syllables into feet. In metrical theory (e.g., Hayes, 1995) it is assumed that strong and weak syllables are grouped to either trochaic or iambic feet in which trochaic feet bear stress on the first syllable and iambic feet on the second syllable. Cairene Arabic is a trochaic and quantity-sensitive language in which bimoraic feet (consisting of either one heavy or two light syllables) are built from the left edge of a phonological word and in which the rightmost of these feet bears main stress (see Section Metrical Properties of Cairene Arabic for details, and also Hayes, 1995; Watson, 2002). Cairene Arabic is quantity-sensitive in the sense that heavy syllables build monosyllabic feet and light syllables bisyllabic ones. The position of stress varies according to the weight of the syllables and the number of feet. Thus, in contrast to languages with a fixed stress position (like final stress in Turkish; e.g., Kaisse, 1985) stress in Cairene Arabic is predictable by structure.

In order to test the effects of predictability and metrical structure, we performed a study measuring EEGs [and calculating event-related potentials (ERPs)] while native speakers of Cairene Arabic listened to correctly and incorrectly stressed words. Such a stress manipulation paradigm in an ERP study has also been applied in studies of German (Domahs et al., 2008), a language with word stress depending on metrical structure, and Turkish (Domahs et al., 2013) with mostly predictable stress. The results of both studies provide starting points to compare stress processing in a language with predictable stress (Turkish) and a language with non-predictable stress guided by metrical structure (German) with Cairene Arabic, in which stress is assumed to be predictable as well as guided by structure. This selection of languages allows us to investigate whether the representation and processing of stress in Cairene Arabic depends mainly on the presence or absence of lexical stress specifications, on metrical structure of words or on both.

## Previous ERP studies on word stress processing

For German and Turkish word stress perception, a series of ERP experiments was performed in which participants were confronted with correctly and incorrectly stressed words of their native language (Knaus et al., 2007; Domahs et al., 2008, 2013).

The measurement of event-related potentials is suitable to investigate the online processing of certain language structures or manipulations in comparison to another condition. ERPs that are obtained via averaging processes over stimuli of the same kind and over participants are negative or positive going deflections time-locked to the stimulus onset and reflecting certain cognitive processes.

In ERP experiments on German or Turkish stress perception, trisyllabic monomorphemic words were presented auditorily, once with the correct stress pattern, and twice with the incorrect ones. The participants' task was to decide whether stress was assigned to the appropriate syllable by pressing either a “yes” or a “no” response button. The visual presentation of the target words, which immediately preceded the auditory input, helped to avoid lexical search effects, and in consequence, facilitated the decision by reducing efforts in lexical retrieval. Furthermore, the visual presentation triggered an expectation that was either met or violated in the auditory stimuli. The studies on Turkish and German demonstrated particular ERP findings, which will be summarized briefly in the following two sections.

### Turkish

Turkish is a language with a clear default pattern: default stress is, according to many descriptions, realized on the word-final syllable (e.g., Lewis, 1967/2000; Sezer, 1981; Hayes, 1995; Kornfilt, 1997; Inkelas, 1999; Kabak and Vogel, 2001, 2011; Inkelas and Orgun, 2003; Göksel and Kerslake, 2005). The regular word-final stress pattern is quantity-insensitive, and long vowels do not attract main stress.

For the study on Turkish prosodic processing (reported in Domahs et al., 2013), a set of words with predictable final stress (e.g., *mıknaˈtız*; “magnet”) and with exceptional lexical stress on the penultimate syllable (e.g., *tiˈyatro*; “theater”) was presented with either correct stress or manipulated stress on each of the other two syllables (e.g., ^*^ˈmıknatız or ^*^mıkˈnatız for words with correct final stress and ^*^ˈtiyatro or ^*^tiyaˈtro for words with correct prefinal stress). Comparisons of stress violations with correct stress conditions revealed that incorrect penultimate stress (e.g., ^*^mıkˈnatız) evoked a positivity (between 850 and 1100 ms), while no such component occurred for the perception of items with incorrect final stress (= default stress) in words with lexical penultimate stress (e.g., ^*^tiyaˈtro).

Such positivity effects in evaluation tasks have been suggested to reflect sensitivity to a deviant structure with an amplitude being correlated with the degree of abnormality (e.g., Picton, 1992; Coulson et al., 1998): The less likely a metrical structure the more pronounced the positivity effect. In the literature, this task-related component has been labeled P300 (e.g., Picton, 1992; Coulson et al., 1998), P600 (e.g., Marie et al., 2011; Schmidt-Kassow et al., 2011a,b) or LPC (e.g., Rugg and Nagy, 1989). The P300 reflects decision-making processes where the reduction of the amplitude indicate that stimulus information is not clear enough. Thus, this component reflects indirectly the grammaticality in stimulus categorization (e.g., Niewenhuis et al., 2005).

**Figure 1:**
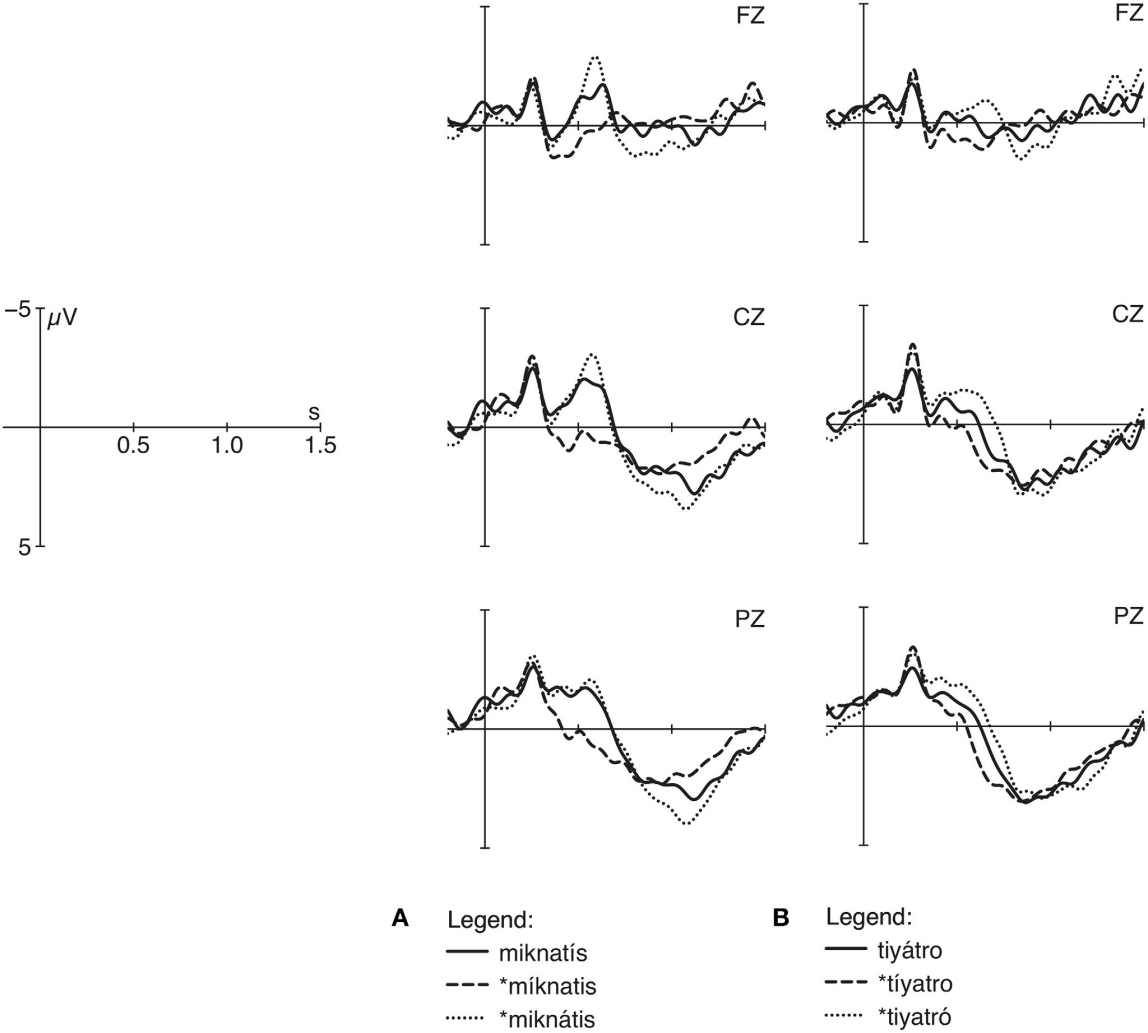
**Grand-average curves of correctly and incorrectly stressed Turkish words (see also Domahs et al., 2013) measured at midline electrodes**. Correct words are illustrated by solid lines, incorrect antepenultimate stress pby dashed lines and incorrect penultiamte/final stress by dotted lines.

The different ERP results for deviating stress patterns in Turkish is depicted in Figure 1. In words with correct final stress (Figure 1A), both violations produce a late positivity if compared with the correct condition. The latency of the positivity, however, differs due to the fact that the position of stressed syllables, which are decisive for the identification of stress patterns, varies. In contrast to Figures 1A,B depicts a positivity effect for violations with initial stress in words with canonical penultimate stress, but no positivity effect for violations involving final stress. The asymmetrical patterning of positivity effects for the two word sets suggests that Turkish participants are sensitive to lexical stress patterns but insensitive to default stress, because violations with lexical stress patterns are perceived as less likely in contrast to violations with the default stress. Thus, our findings support and complement findings by Peperkamp et al. (2010) for languages with predictable stress.

In addition to the P300 effect, an N400 effect, a negative going deflection around 250 and 500 ms post-stimulus onset, was obtained for violations with final stress. This effect was interpreted to reflect brain responses to an unexpected stimulus that produce higher costs in lexical retrieval (for a review of the N400 component see Kutas and Federmeier, 2011). Note that a shift from the lexical non-final stress position (tiˈyatro) to the default (^*^tiyaˈtro) involves a violation of a lexical stress specification. It is most remarkable that the Turkish participants showed this negative deflection mirroring the violation of an expected stress pattern while they had difficulties to classify the incorrect default stress as violating. The difficulties were not only indicated by a lack of a P300 effect but also by high error rates in the behavioral data.

### German

German monomorphemic words allow for final, penultimate, or antepenultimate stress. Which pattern to occur cannot be adequately predicted by means of stress rules. Though the stress position itself is considered not predictable, the underlying prosodic structure can be determined mostly on the basis of the weight of the final syllable. In most accounts of German phonological words, trochees are built in a right-to-left manner (Eisenberg, 1991; Wiese, 1996; Féry, 1998; Janssen, 2003). In words with a heavy final syllable (Vitamin—((vi.ta)_F_(mi:n)_F_)_ω_), the final syllable constitutes a non-branching foot (a moraic trochee), and in words with a light final syllable, the final syllable constitutes the weak syllable of a bisyllabic trochee. Thus, trisyllabic words varying in the structure of the final syllable consist of either two feet ((σσ)_F_(σ)_F_)_ω_ or one foot (σ(σσ)_F_)_ω_ (for such an analysis see Janssen, 2003; Domahs et al., 2008, 2014; Knaus and Domahs, 2009; Röttger et al., 2012).

**Figure 2:**
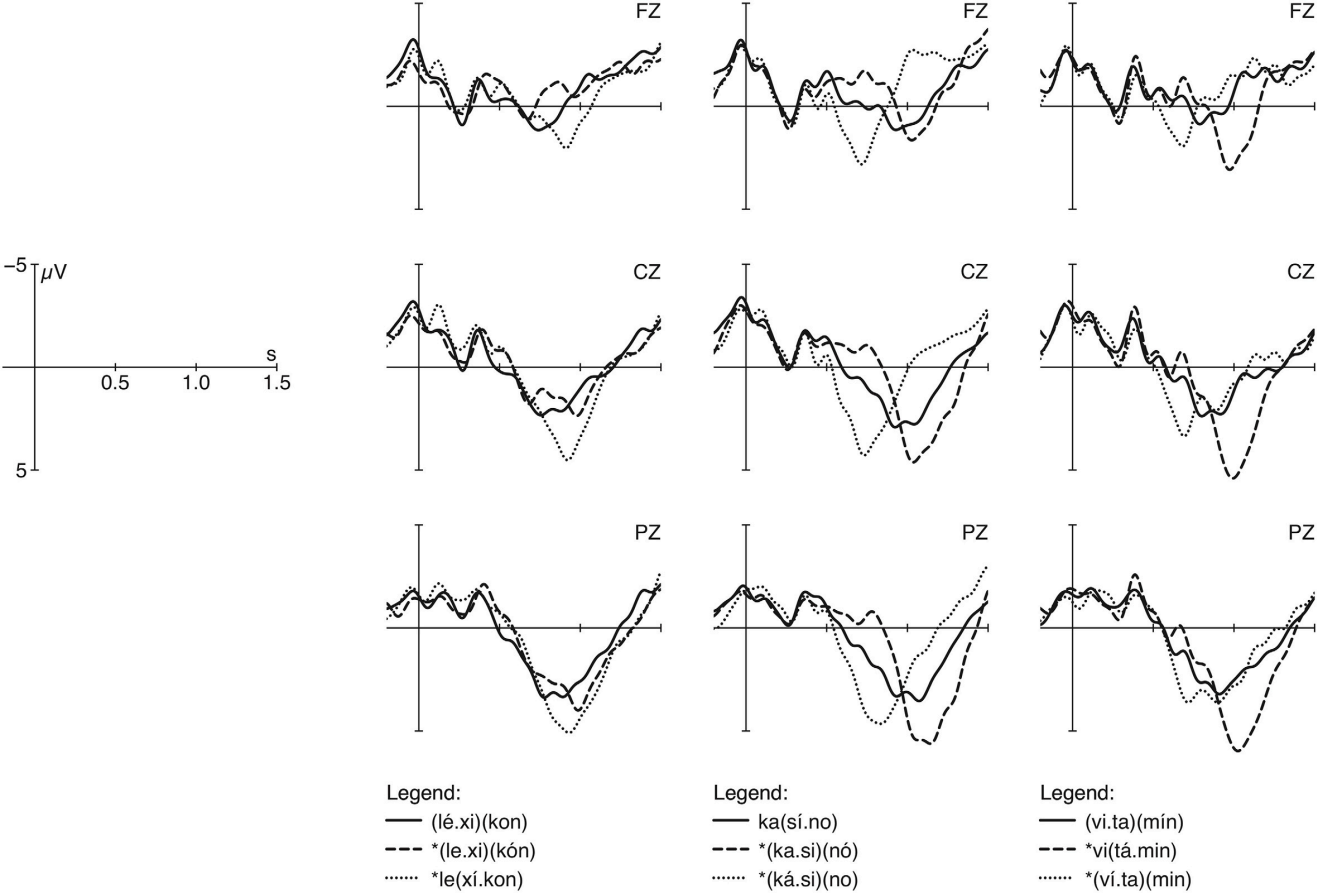
**Grand-average curves of correctly and incorrectly stressed German words (see also Domahs et al., 2008) measured at midline electrodes**.

The experiment on German word stress evaluation (reported in Domahs et al., 2008) revealed different ERP patterns compared to the findings on Turkish. Again words with antepenultimate, penultimate and final stress were recorded with correct and deviating stress on each of the other two syllables. In contrast to Turkish, no effect of default stress was found, although several proposals assume penultimate stress to be the default stress pattern (as in *Kaˈsino*; “casino”) in German. If the penultimate stress were the default stress pattern, we would expect this pattern not to evoke a late positivity when used incorrectly. However, incorrect penultimate stress in trisyllabic words with either correct final (e.g., ^*^Viˈtamin instead of *Vitaˈmin*; “vitamin”) or initial stress (e.g., ^*^Leˈxikon instead of *ˈLexikon*; “lexicon”) evoked enhanced positivity effects (between 900 and 1150 ms) showing that participants can decide clearly that this stress is incorrect (see Figure 2). However, comparisons between correct and incorrect conditions revealed another form of asymmetric results regarding the occurrence or non-occurrence of a P300 component in German stress perception: stress violations produce enhanced positivity effects whenever the stress derivation leads to a change in foot structure (e.g., ^*^vi(ˈ*ta.min*)_F_ instead of (*vi.ta*)_F_(ˈ*min*)_F_), but not if the foot structure is maintained (e.g., ^*^(ˈ*vi.ta*)_F_(*min*)_F_ instead of (*vi.ta*)_F_(ˈ*min*)_F_). In contrast to Turkish, it is not the main stress position but rather the internal prosodic structure of words that is more or less predictable and has an impact on the processing of word stress (see Janssen, 2003; Domahs et al., 2014). In addition, behavioral data (error rates) indicate that German participants are sensitive to stress manipulations and identify incorrect stress with high accuracy, while Turkish participants recognized violations involving default stress at chance level only.

In the present paper, we examine a third type of language, Cairene Arabic, with a predictable and foot based stress system. Strictly bimoraic feet are built from left to right and the rightmost foot receives main stress (see below Section Metrical Properties of Cairene Arabic). Hence, Cairene Arabic is situated between the Turkish and German system by having predictable word stress like Turkish, but varying positions of word stress due to quantity sensitive foot formation like German. The main goal was to see whether speakers of Cairene Arabic are insensitive to the very predictable stress positions in their language (as Turkish participants have been shown to be insensitive to the predictable stress pattern), or whether asymmetrical ERP results occur along the lines of metrical structure (stress derivation that change the structure produce P300 effects and those that maintain structure not). To test this, trisyllabic words with penultimate and final stress were compared in two conditions each: (i) penultimate words with one foot [e.g., *va*(ˈ*nil*)_F_*ja*; “vanilla”; in the following word type 1] and with two feet [e.g., (*mus*)_F_(ˈt*a*ʃ)_F_*fa*; “hospital”; in the following word type 2] and (ii) finally stressed words with a bisyllabic initial foot and a monosyllabic final foot [e.g., (*vi.ta*)_F_(ˈ*mi:n*)_F_; in the following word type 3] and with two monosyllabic feet [e.g., *ki*(*ris*)_F_(ˈ*ta:l*)_F_“crystal”; in the following word type 4]. If structure licenses stress positions, we should find that deviating stress realized on a strong syllable of a foot produces less pronounced positivities compared to deviating stress on a weak syllable (for instance, incorrect antepenultimate stress in words of the structure (*mus*)_F_(ˈt*aʃ*)_F_*fa* should evoke less pronounced effects compared to incorrect antepenultimate stress in words of the structure *va*(ˈ*nil*)_F_*ja*).

Before we continue to present the experiment on Cairene Arabic we would like to introduce the main characteristics of the Cairene Arabic stress system.

## Metrical properties of Cairene Arabic

The Cairene Arabic dialect of Arabic is the most widely spoken language in Egypt. Half of the population speaks the Cairene Arabic dialect as its first language. Note that Cairene Arabic is a spoken language (though also written forms exist), while the literary language of Egypt is Standard Arabic (Woidich, 2006).

Cairene Arabic is not only the most widely spoken dialect in Egypt, it is also the best described Arabic dialect, particularly as regards its metrical structure. In the literature, pre-generative (Harrell, 1960; Mitchell, 1960), generative (Hayes, 1995; Watson, 2002), and typological accounts (Hulst van der and Hellmuth, 2010) exist, which all identify Cairene Arabic as a quantity-sensitive language in which the parsing of syllables into feet is sensitive to syllabic weight: a super-heavy final syllable with long vowels followed by a consonant (CVVC) receives main stress, otherwise a heavy penult with a long vowel or a short vowel followed by a consonant is stressed or a light antepenult in words ending in three light syllables (open syllables with short vowels). According to McCarthy (1979), bimoraic trochees consisting of either one heavy syllable or two light syllables are built in a left to right manner. In (1) examples for words with final, penultimate, and antepenultimate stress are given.

(1)
  - **final stress**
    [gaˈto:] “cake”, [vitaˈmi:n] “vitamin”, [kirisˈta:l] “cristal”
  - **penultimate stress**
    [ˈbe:tak] “your house”, [vaˈnilja] “vanilla”, [musˈta ʃ fa] “hospital”
  - **antepenultimate stress**
    [ˈkazino] “casino”, [sanˈtimitir] “centimeter”

The syllable in Cairene Arabic consists obligatorily of a single onset consonant followed by a short or long vowel. The coda maximally includes two consonants, but only one consonant in word-medial position. Syllable weight is important for the foot formation in Cairene Arabic because feet consist of minimally and maximally two moras, a unit proposed to define syllable weight (e.g., Hyman, 1985). Accordingly, syllables with a long vowel or a short vowel followed by a coda consonant (two moras) are heavy and syllables with a short vowel (one mora) are light. For word final syllables different conditions must be met for a syllable to be heavy because the final consonant is analyzed to be extrametrical, i.e., does not contribute to syllable weight. Therefore, a final syllable is heavy if it consists of a long vowel or a short vowel followed by two consonants. These properties of heavy and light syllables guide the foot formation of phonological words in Cairene Arabic. In (2), the analysis according to Hayes (1995: 69/70; following McCarthy, 1979) is summarized.

(2) Rules for Cairene Arabic stress
  - word final consonants are extrametrical: C → <C> / ___]_word_
  - foot construction: Build up bimoraic trochaic feet from left to right
    No degenerate feet!
  - word layer construction: Group feet into a right-headed word constituent
    (End Rule Right)

We also note that there are other types of evidence for the bimoraic trochee in this language although secondary stress corresponding to a foot not carrying word stress has been reported to be absent (Watson, 2002, ch. 5): the word in Cairene Arabic minimally consists of a bimoraic foot. Furthermore, there is a productive pattern for nick names or hypocoristics in which names of any prosodic shape are truncated to a bimoraic foot, see examples in (3).

**Table:** 

(3)	**full form**	**hypocoristic**
	Fahd	ˈdo.do
	Karim	ˈKi.ki
	Shaimaa	ˈʃo.ʃo
	Mostafa	ˈSˁa.sˁa

The present study is designed to investigate whether the foot structure as proposed in metrical analyses of Cairene Arabic are psychologically real and used during the processing of lexical words.

## ERP experiment on Cairene Arabic

The method used in the present ERP-experiment was adopted from the ones on German and Turkish reported in Domahs et al. (2008) and Domahs et al. (2013). Similar to the previous studies, participants were confronted with correctly and incorrectly stressed words and instructed to judge the correctness of the stress patterns. Given the results on German, this stress-violation paradigm utilizing explicit judgments of stress proved to be suitable to investigate factors involved in prosodic processing of words. In particular, this method enables to identify potential stress positions irrespective of the correct one. In the following, we will present the experiment on Cairene Arabic in more detail and compare the results with those obtained from German and Turkish participants.

### Cairene Arabic

The aim of the present experiment is to test whether (i) native speakers of Cairene Arabic are sensitive to stress manipulations and (ii) whether the processing of stress manipulations is influenced by foot structure. For this purpose, participants were presented with correctly and incorrectly stressed trisyllabic words differing in syllable and foot structure.

#### Participants

Twenty-three right-handed native speakers of Cairene Arabic (20 men) were recruited for participation at the University Marburg, all of which having normal or corrected-to-normal vision and no hearing deficits. The participants' age ranged from 26 to 45 (mean age 32). All participants were born and raised monolingually in and around Cairo in Egypt, all from the Cairene Arabic dialect region. The participants' language skills comprised of second language knowledge of English, German, French, or Spanish. All participants stated to have been raised monolingually with Cairene Arabic as ambient language, and had been in Germany for 36 month in mean before participation, ranging from 1 month up to 7 years. Participants were instructed in Cairene Arabic to ensure that participants are well informed. Each participant was paid for his/her contribution. The data sets of three participants had to be excluded due to missing responses, left-handedness or excessive movement artifacts.

Note that a balanced proportion of women and men could not be obtained due to the fact that participation would have required removing the headscarf.

#### Material

In order to be able to investigate whether the foot structure constrains the processing of stress shifts, we investigated four word types that different in foot structure, as summarized in Table 1. Words with structure 1 and 2 are canonically stressed on the penultimate syllable and consist of heavy penultimate syllables with either long or short vowels followed by a consonant (for the sake of clarity only rhyme structures are illustrated, i.e., a structure CVC is mentioned as VC) and the first syllable is either footed or not, words with structure 3 and 4 are canonically stressed on the final syllable and contain super heavy final syllables. In structure 3, the first two syllables constitute a bisyllabic foot while in structure 4 the heavy penult constitutes a monosyllabic foot.

**Table 1:** **Conditions and material**.

**Structure**	**Conditions**	**Examples**	
1	Correct PU stress	va.ˈnil.ja	“vanilla”
V(VC)V	Incorrect APU stress	*ˈva.nil.ja	
	Incorrect final stress	*va.nil.ˈja	
2	Correct PU stress	mus.ˈtaʃ.fa	“hospital”
(VC)(VC)V	Incorrect APU stress	*ˈmus.taʃ.fa	
	Incorrect final stress	*mus.taʃ.ˈfa	
3	Correct final stress	vi.ta.ˈmi:n	“vitamin”
(V.V)(VVC)	Incorrect APU stress	*ˈvi.ta.mi:n	
	Incorrect PU stress	*vi.ˈta.mi:n	
4	Correct finals stress	ki.ris.ˈta:l	“crystal”
V(VC)(VVC)	Incorrect APU stress	*ˈki.ris.ta:l	
	Incorrect PU stress	*ki.ˈris.ta:l	

In words with canonical penultimate stress (structure 1 and 2), the question is whether stress moved from penultimate syllable to antepenultimate syllable produce less pronounced P300 effects when the antepenultimate syllable is head of a foot (structure 2) in comparison to unfooted (structure 1). In words with canonical final stress (structure 3 and 4), either the antepenultimate syllable (structure 3) or the penultimate syllable (structure 4) is the head of a foot and therefore a potential landing site for stress. Though the existence of secondary stress is disputed in Cairene Arabic, the question arises whether words are exhaustively parsed into feet and whether heads of feet are stressable in contrast to weak syllables of feet.

For each type of trisyllabic words, a set of 15 monomorphemic items (as given in Appendix) was selected and recorded by a female native speaker of Cairene Arabic in a sound-proof booth (44 kHz, 16 bit, mono). Each word was realized in the correct and in the two incorrect conditions (see Table 1). In order to ensure that incorrect stresses were not produced in an exaggerated manner, correct and incorrect words with the same stress pattern were recorded in a randomized list. The phonetic parameters of duration, intensity, and F0 of each stress pattern were compared between correct and incorrect conditions (e.g., between correct *kirisˈta:l* and incorrect ^*^vanilˈja, see Table 2 with mean values for each stress patterns) showing that incorrect and correct stress realizations of a certain stress pattern differ significantly only with respect to duration because correct and incorrect conditions differ in syllable structure (e.g., *kirisˈta:l* ends in a super heavy syllable while ^*^vanilˈja does not; for the statistical analyses of phonetic parameters see Table 2). But crucially, correct and incorrect versions of each stress pattern do not differ regarding F0 and intensity.

**Table 2:** **Mean values (SD in parentheses) of phonetic parameters fundamental frequency (F0 in Hz), duration (ms), and intensity (dB) as well as repeated measures ANOVAs on the factor correctness (correct vs. incorrect) per stress pattern**.

**Stress pattern**	**Condition**	**Parameter**	**1st syllable**	**2nd syllable**	**3rd syllable**
Antepenultimate stress	Correct (filler items)	F_0_	238 (8.7)	204 (6.0)	168 (29.9)
		Duration	226 (55)	196 (33)	278 (55)
		Intensity	53.3 (3.3)	51.2 (4)	39.4 (3.7)
	Incorrect	F_0_	238 (11.0)	199 (9.3)	180 (25.6)
		Duration	279 (80)	233 (51)	300 (64)
		Intensity	53.3 (5)	47.4 (5.5)	39.5 (4.2)
Penultimate stress	Correct	F_0_	216 (5.6)	228 (9.6)	189 (15.5)
		Duration	265 (68)	358 (67)	339 (52)
		Intensity	49.1 (4.9)	55.7 (3.6)	41.6 (3.2)
	Incorrect	F_0_	219 (11.3)	231 (7.5)	183 (25.9)
		Duration	216 (62)	425 (62)	386 (81)
		Intensity	51.2 (6.5)	51.8 (4.7)	40.1 (3.9)
Final stress	Correct	F_0_	221 (8.0)	218 (5.2)	214 (7.2)
		Duration	172 (57)	255 (68)	629 (73)
		Intensity	52 (4.5)	52 (5.9)	48.6 (3.5)
	Incorrect	F_0_	218 (9)	216 (5)	215 (5.9)
		Duration	224 (78)	232 (63)	539 (77)
		Intensity	50.7 (5.3)	49.7 (5.7)	48.4 (3.4)
**REPEATED MEASURES ANOVA**					
Antepenultimate stress		F_0_	*F*_(1, 19)_ = 2.49; *p* > 0. 13		
		Duration	*F*_(1, 19)_ = 15.4; *p* < 0.001***		
		Intensity	*F*_(1, 19)_ = 3.2; *p* > 0.08		
Penultimate stress		F_0_	*F*_(1, 29)_ < 1		
		Duration	*F*_(1, 29)_ = 11.0; *p* < 0.003**		
		Intensity	*F*_(1, 29)_ < 1		
Final stress		F_0_	*F*_(1, 29)_ = 2.3; *p* > 0.14		
		Duration	*F*_(1, 29)_ = 8.4; *p* < 0.008**		
		Intensity	*F*_(1, 29)_ < 1		

Significant results are indicated by <*>

Significant results at 1% level are indicated by <**> and at 0.1-level by <***>.

Furthermore, the stimuli were not spoken in isolation but embedded in the following carrier sentence:

(3) howa lazem ye?ool **vitami:n** delwa?ti “He has to say Vitamin now!”

The carrier sentence was identical for each critical stimulus and included the stimulus in a citation-like context bearing nuclear stress. The carrier sentence avoids a list reading and a pitch fall at the end of the critical words.

Each of the 15 items per word condition was presented in the correct and in the two incorrect conditions. To increase the number of items per condition, each version of a stimulus was presented twice. Thus, the total number of critical items was 4 (word types) × 15 (individual items) × 3 (stress patterns) × 2 (repetitions) resulting in 360 tokens. In addition, 80 trials including words with correct antepenultimate stress were included as filler. This was done to ensure that each stress pattern occurred in correct and incorrect conditions, and that the number of correctly and incorrectly stressed words was balanced.

#### Procedure

Participants were seated in front of a computer screen in a sound-proof room. In each trial they were confronted with the visual presentation of an experimental item followed by the auditory presentation of the same item. The participants' task was to decide as accurately as possible whether the auditory stimuli were correctly stressed or not by pressing a response key of a push-button box. The task required the participants to activate internal stress representations (from the written input) and to compare these representations with stress information in the auditory presentation.

Each trial started with a fixation cross that appeared for 500 ms. An experimental item was then presented visually for 900 ms, followed by a blank screen for 250 ms before the auditory presentation of the stimulus started. The mean duration of the sentences was 3.9 seconds. Throughout the auditory presentation, the participants were asked to fixate on a cross in the center of the screen to avoid eye movement artifacts while listening. After the offset of each sentence, a question mark appeared on the screen and remained there until a yes or no button was pressed with a timeout of 2000 ms. Responses were given after the appearance of the question mark, but not immediately while listening to the critical items, to avoid movement artifacts. The assignment of thumbs to the yes and no buttons was counterbalanced across participants. During the answering period and the following intertrial interval of 3000 ms, the participants were allowed to blink and to rest their eyes. The experiment was controlled by the Presentation software (Version 15; Neurobehavioral Systems).

The stimuli appeared in eight experimental blocks consisting of 55 stimuli each, preceded by a short practice phase. Experimental and filler items were presented in pseudo-randomized order, each word appearing only once within each block. The order of blocks was varied for each participant to avoid sequence effects. The entire duration of the experimental session was approximately 60 min.

#### Data acquisition and analyses

**(a) Behavioral Data**

During each trial accuracy and reaction time data were measured. For statistical analyses, only the accuracies of judgments were calculated because response latencies were measured after the offset of the sentences with a delay of approximately 880 ms. The accuracy scores were calculated for each participant and condition and for each stimulus and condition.

In two repeated measures ANOVAs, the factors foot structure (two different structures) and stress position (antepenultimate, penultimate, and final) were analyzed in a 2 × 3 design for words with canonical penultimate and canonical final stress separately. We calculated two separate ANOVAs due to the fact that the structure conditions for words with either penultimate or final stress vary systematically.

**(b) ERP Data**

An electroencephalogram (EEG) was recorded from overall 24 Ag/AgCl electrodes via a *BrainVision* (Brain Products) amplifier. Four electrodes measured the electro-oculogram (EOG), i.e., horizontal and vertical eye movements. The reference electrode was placed at the left mastoid. EEGs were re-referenced off-line to both mastoids. The C2 electrode served as ground. The head electrodes were mounted on an elastic cap (Easy Cap). EEG and EOG were recorded with a sampling rate of 500 Hz and filtered offline with a 0.3 to 20 Hz bandpass filter. All electrode impedances were kept below 5 kΩ. Prior to data analysis, all individual EEG recordings were automatically and manually scanned for artifacts from eye or body movements and muscle artifacts. In total, 7.5% of the data with an amplitude change of more than 40 μV had to be excluded from analysis.

Averages were calculated per participant and condition starting from the onset of the auditory stimulus up to 1500 ms. For words with correct penultimate or final stress, incorrect conditions were compared with correct conditions. In analogy to earlier studies (Domahs et al., 2008, 2013), time-windows were chosen by visual inspection for the two sets of words with canonical penultimate and final stress pattern separately because the latency of effects reflecting the evaluation of stress patterns and decision making seem to depend on the position of the stressed syllable. Therefore, effects measured for words with incorrect antepenultimate stress occur earlier than effects found for incorrect penultimate and final stress.

Furthermore, violations with penultimate and final stress evoked a biphasic pattern consisting of a negativity followed by a positivity, while violations with antepenultimate stress evoked only a positivity. This lack of a negativity is due to the fact that the positivity occurs within the negativity time-window. Table 3 provides an overview of time-windows per word type and incorrect stress condition. For each time window, a general analysis of variance with repeated measures (ANOVA) was calculated for words with canonical penultimate and canonical final stress separately over the factors foot structure (the two different foot structures per correct stress pattern; structure 1 and 2 are compared for words with canonical penultimate stress and structure 3 and 4 for words with canonical final stress) correctness (correct vs. incorrect) and region (frontal, central, parietal). Region is defined as a three-level factor with the values frontal (including F3, Fz, F4), central (including C3, Cz, C4), and parietal (including P3, Pz, P4).

**Table 3:** **Time-windows for statistical analyses**.

**Stress condition**	**Structure**	**Violation type**	**Time-windows**	
			**Negativity effect**	**Positivity effect**
Correct penultimate stress	1	Antepenultimate stress	–	350–600 ms
	V(ˈVC)V	Final stress	400–550 ms	800–1150 ms
	2	Antepenultimate stress	–	350–600 ms
	(VC)(ˈVC)V	Final stress	400–550 ms	800–1150 ms
Correct final stress	3	Antepenultimate stress	–	300–650 ms
	(V.V)(ˈVVC)	Penultimate stress	400–480 ms	550–850 ms
	4	Antepenultimate stress	–	300–650 ms
	V(VC)(ˈVVC)	Penultimate stress	400–480 ms	550–850 ms

*For each word type, the correct conditions with two different foot structures are compared to each incorrect condition. Time windows are given for negativity and positivity effects*.

#### Results

**(a) Behavioral Data**

**Figure 3:**
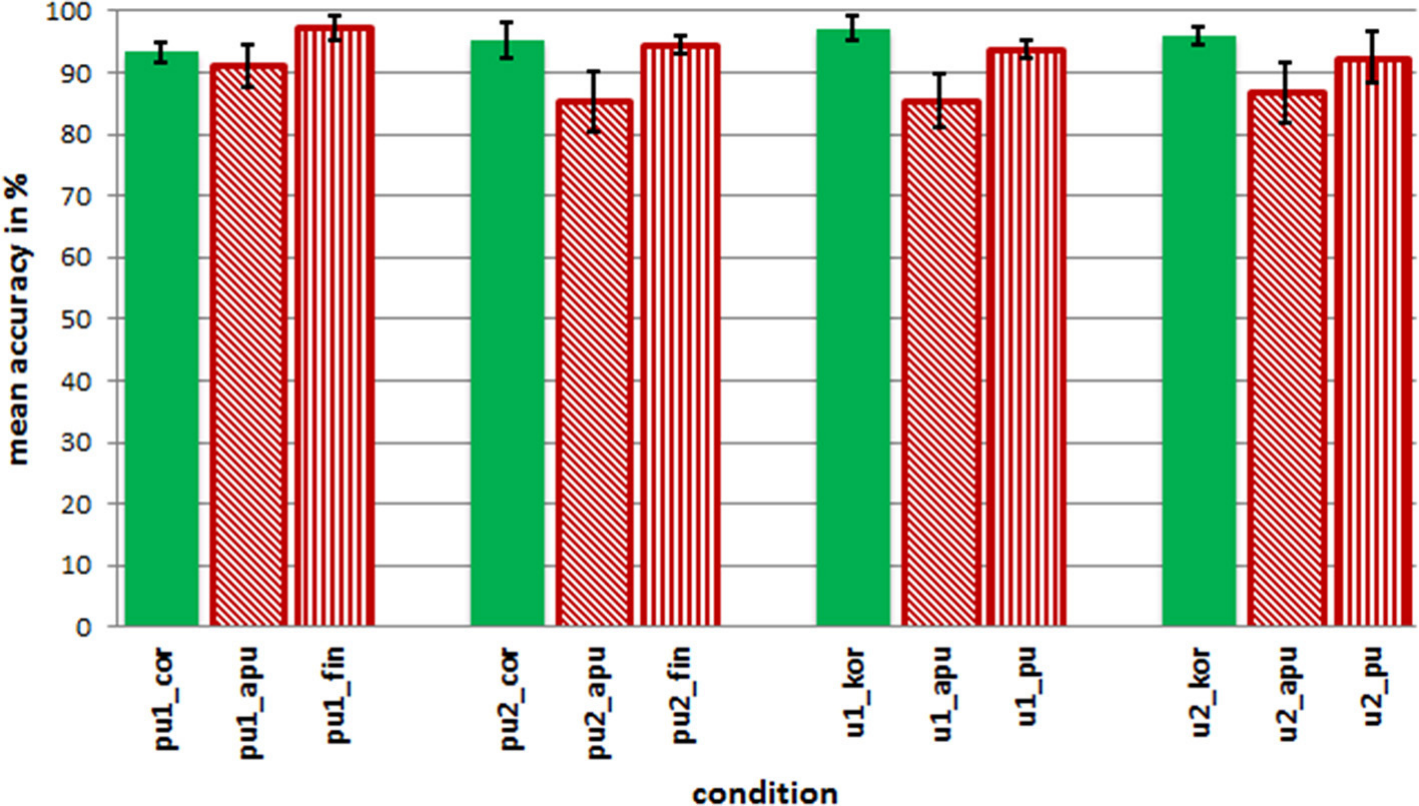
**Mean accuracy in percent for each word type and condition**.

In the analyses of accuracy scores, the aim was to investigate whether specific conditions were more error-prone than others. A repeated measures ANOVA of arcus-sinus transformed accuracy scores was calculated over the factors foot structure (two different structures) and stress position (antepenult, penult, and final stress) for the two sets of words with either canonical penultimate or final stress, and pairwise *t*-tests comparing correct with incorrect stress and both incorrect conditions per word set. Figure 3 depicts the mean accuracy scores for all conditions.

Generally, speakers of Cairene Arabic are accurate with their judgments for more than 80% in each condition. This finding suggests that they are in principle sensitive to the presented stress manipulations. However, the accuracy for all conditions differs slightly, and as is illustrated in Figure 3, the mean accuracy for conditions with incorrect antepenultimate stress is lower compared to other conditions. Repeated measures ANOVAs and paired *t*-tests are calculated for words with canonical penultimate and final stress separately (see Table 4).

**Table 4:** **Statistical analyses of behavioral data**.

**Analysis**		**Results**
**ANOVA FOR WORDS WITH CANONICAL PENULTIMATE STRESS**		
Foot structure		*F*_(1, 19)_ = 5.45; *p* < 0.04*; pes.223
Stress position		*F*_(2, 38)_ = 7.59; *p* < 0.005**: pes.285
Interaction of foot structure × stress position		*F*_(2, 38)_ = 8.06; *p* < 0.004**; pes.298
**PAIRWISE *t*-TESTS**		
Word type 1 V(ˈVC)V	Correct vs. antepenultimate stress	*t*_(19)_ = −0.25; *p* > 0.80
	Correct vs. final stress	*t*_(19)_ = −2.26; *p* < 0.04
	Antepenultimate vs. final stress	*t*_(19)_ = −3.56; *p* < 0.003**
Word type 2 (VC)(ˈVC)V	Correct vs. antepenultimate stress	*t*_(19)_ = −1.79; *p* > 0.08
	Correct vs. final stress	*t*_(19)_ = −2.38; *p* < 0.03
	Antepenultimate vs. final stress	*t*_(19)_ = −7.78; *p* < 0.001***
**ANOVA FOR WORDS WITH CANONICAL FINAL STRESS**		
Foot structure		*F*_(1, 19)_ < 1
Stress position		*F*_(2, 38)_ = 5.36; *p* < 0.02*; pes = 0.22
Interaction of foot structure × stress position		*F*_(2, 38)_ = 6.72; *p* < 0.004**; pes = 0.261
**PAIRWISE *t*-TESTS**		
Word type 3 (V.V)(ˈV:C)	Correct vs. antepenultimate stress	*t*_(19)_ = −1.96; *p* > 0.06
	Correct vs. penultimate stress	*t*_(19)_ = 1.02; *p* > 0.32
	Antepenultimate vs. penultimate stress	*t*_(19)_ = −5.14; *p* < 0.001***
Word type 4 V(VC)(ˈV:C)	Correct vs. antepenultimate stress	*t*_(19)_ = −2.53; *p* < 0.02
	Correct vs. penultimate stress	*t*_(19)_ = −0.95; *p* > 0.35
	Antepenultimate vs. penultimate stress	*t*_(19)_ = −2.89; *p* < 0.01

Repeated measures ANOVA for the two sets of words with canonical penultimate and final stress separately over the factors foot structure and stress position as well as pairwise t-tests for comparisons of correct with each of the two incorrect stress conditions and of both incorrect conditions. According to Bonferroni correction, the level of significance for paired t-tests is below 0.008. Significant results are indicated by < * >. Effect sizes are given by partial Eta-squared values (pes).

Analyses for words with correct penultimate stress yield a main effect for the factors foot structure and stress position as well as an interaction of both factors. *Post-hoc t*-tests comparing mean accuracies of the correct condition with each incorrect condition and of both incorrect conditions revealed a significant difference between two incorrect conditions. This holds for both word types with canonical penultimate stress.

Analyses for words with correct final stress yield a main effect for the factor stress position and an interaction of the factors foot structure and stress position. *Post-hoc t*-tests revealed a significant difference between mean accuracy for incorrect antepenultimate stress and incorrect penultimate stress in words of the structure (V.V)(V:C) but not in words of the structure V(VC)(V:C). Overall, the analyses suggest that conditions with incorrect antepenultimate stress are more error-prone than correct conditions and other incorrect stress conditions. This could be interpreted as an uncertainty toward words containing incorrect antepenultimate stress. Note that accuracies for correct words with antepenultimate stressed (filler condition) scored high with 98% correct responses.

**(b) ERP Data**

**Figure 4:**
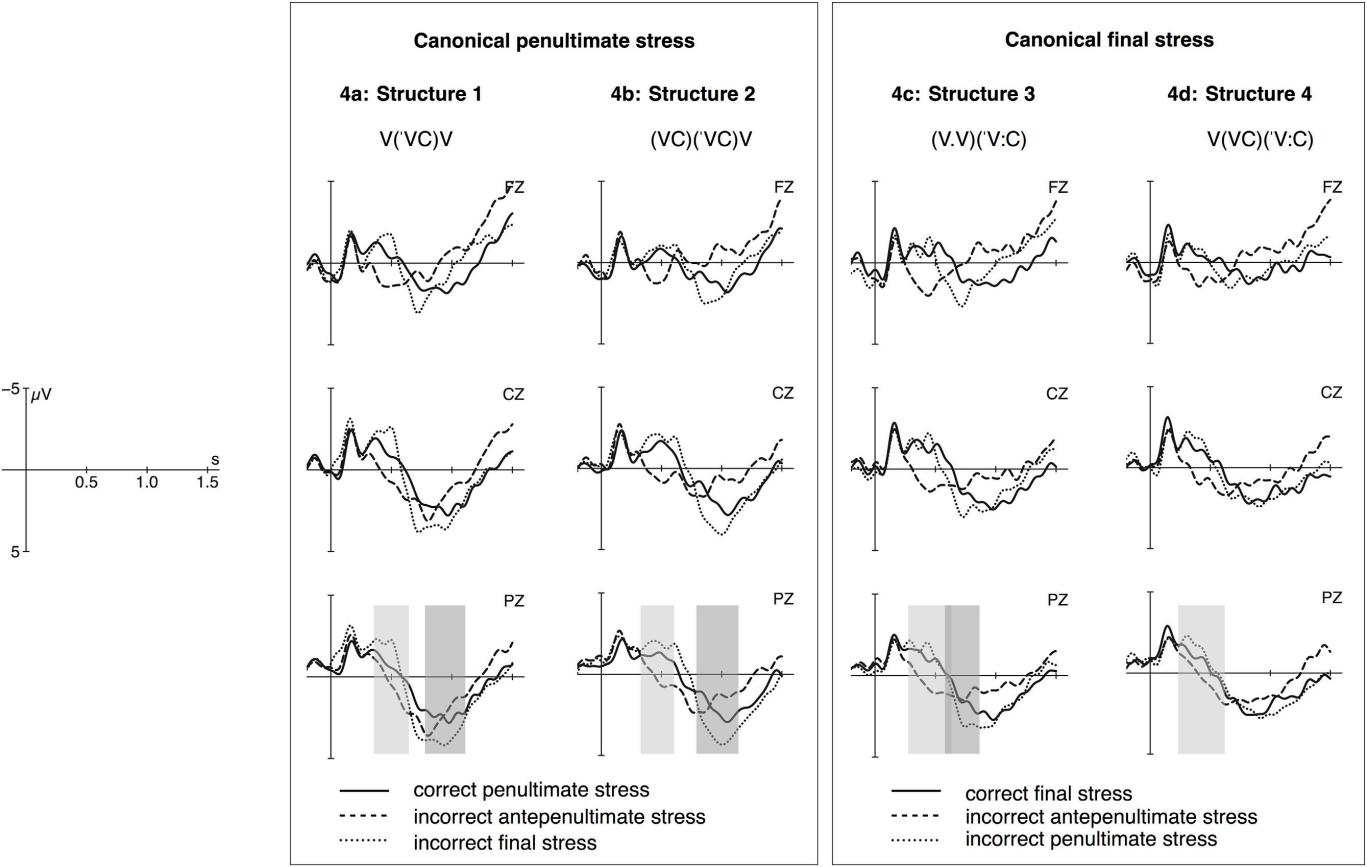
**Grand Averages of event-related potentials (ERPs) measured at midline electrodes for words with canonical penultimate stress with Structure 1 (a) and 2 (b) and canonical final stress with Structure 3 (c) and Structure 4 (d)**. Correct conditions (solid lines) are plotted against the incorrect conditions with antepenultimate stress (dashed lines) and with penultimate/final stress (dotted lines). The light gray bars indicate time-windows for positivity effects evoked by words with incorrect antepenultimate stress and the darker gray bars for positivity effects evoked by words with incorrect penultimate and final stress.

For the analyses of mean voltage changes induced by stress manipulations, we calculated for each set of words with either canonical penultimate or final stress whether each of the two incorrect conditions differ significantly from the correct condition and whether the foot structure influences the processing of incorrectly stressed words. Figure 4 shows the grand averages at midline electrodes for the four word types. Generally, we observed positivity effects for stress deviations involving antepenultimate stress and a biphasic ERP pattern for violations with penultimate or final stress. As noted in Section Data Acquisition and Analyses, effects for violations with antepenultimate stress occur in earlier time-windows compared to effects for violations with penultimate or final stress. Therefore, mean voltage changes for the processing of separate stress deviations were analyzed in different time windows. Appendix provides an overview of statistical analyses. In the following, the results are presented for each set of words with either penultimate or final stress separately.

***Words with canonical penultimate stress***. Violations with antepenultimate stress (dashed line in Figures 4A,B) produced a positivity effect between 350 and 600 ms in the two word types with canonical penultimate stress. A main effect for the factors correctness and region and an interaction for foot structure × correctness × region occurred. *Post-hoc* analyses confirm significant differences between correct and incorrect antepenultimate stress in each region and for each structure (see **Table A2A**).

Violations with final stress in words with canonical penultimate stress (dotted line in Figures 4A,B) evoked a biphasic ERP pattern consisting of a negativity effect between 400 and 550 ms and a positivity effect between 800 and 1150 ms. For the negativity, repeated measures ANOVAs revealed a main effect for the factors correctness and region and an interaction for region × foot structure for which *post-hoc* analyses exhibited no significant structure effects in the three regions (see **Table A2B**). For the positivity effect, a main effect for the factors correctness and region and a three-way interaction was obtained. *Post-hoc* analyses show that mean voltages differ significantly between correct and final stress in parietal region for words of the structure V(VC)V, and in centro-parietal region for words of the structure (VC)(VC)V (see **Table A2C**).

***Words with canonical final stress***. For violations with antepenultimate stress (dashed lines in Figures 4C,D), positivity effects occurred between 300 and 650 ms in both word types with canonical final stress. Repeated measures ANOVAs over the factors foot structure, correctness and region revealed a main effect for the factor correctness and an interaction for correctness × region and correctness × foot structure. *Post-hoc* analyses showed a difference between correct final stress and incorrect antepenultimate stress in each region and each foot structure (see **Table A2D**).

Violations with penultimate stress (dotted lines in Figures 4C,D) led to a negativity effect between 400 and 480 ms and to a positivity effect between 550 and 850 ms only in the context of word type 3 with the structure (V.V)(V:C), but not for word type 4 with a strong penultimate syllable V(VC)(V:C). For the negativity effect, a main effect for all three factors but no interaction was found (see **Table A2E**), and for the positivity a main effect for the factors correctness and region and an interaction between correctness × region as well as correctness × foot structure. *Post-hoc* analyses suggest that an overall effect of correctness is restricted to frontal regions only and that a difference between correct and incorrect penultimate stress occurs only for words of the structure (V.V) (V:C) (see **Table A2F**).

**Figure 5:**
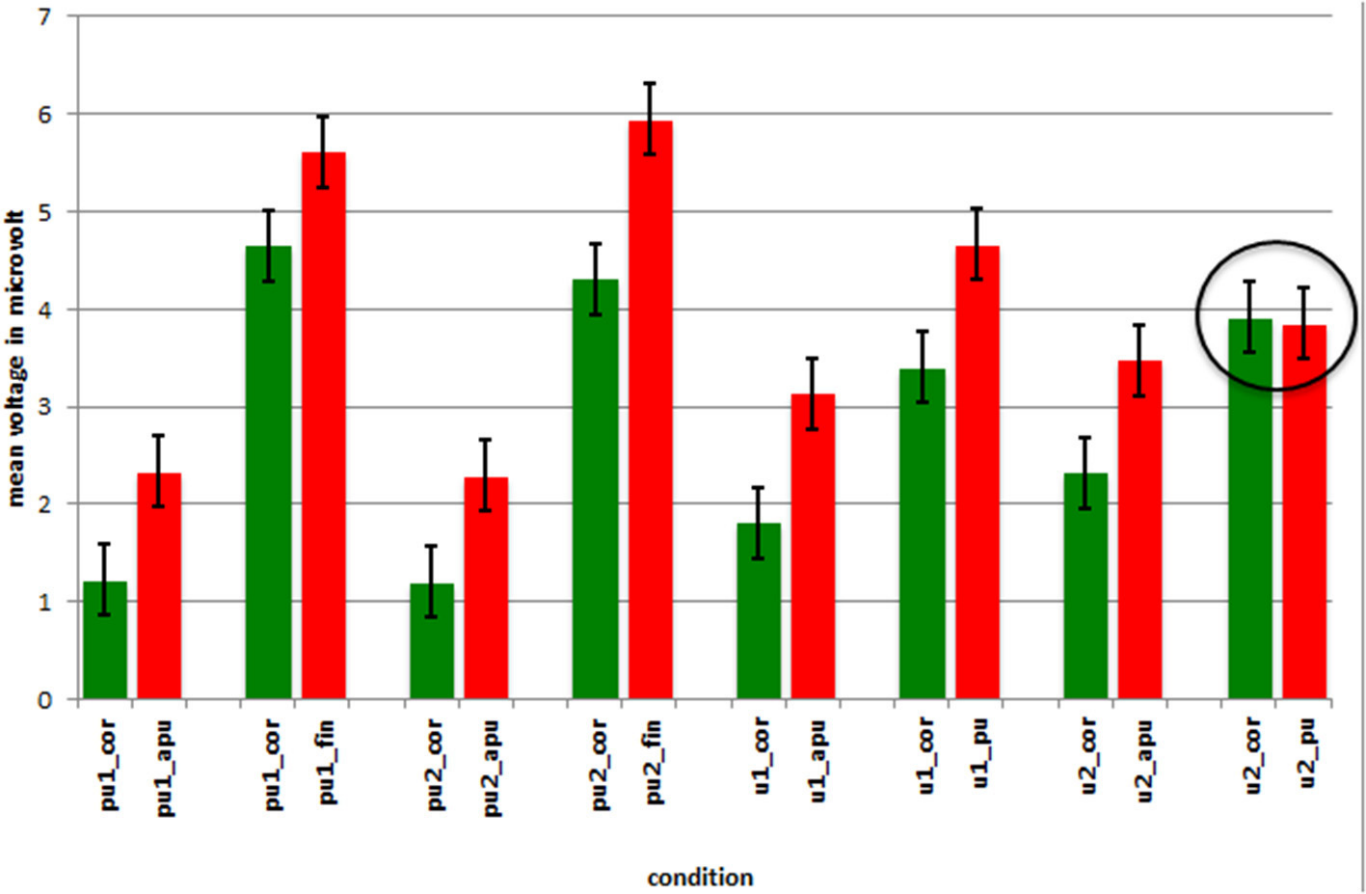
**Mean amplitudes and standard errors in microvolt for positivity effects of correct and incorrect conditions measured at parietal electrodes**. The label pu1 denotes conditions of word type 1, pu2 conditions of word type 2, u1 conditions of word type 3 and u2 conditions of word type 4. The circle indicates the conditions for which the comparison was not significant.

Figure 5 depicts mean amplitudes of respective peaks of positivity effects for correct and incorrect conditions measured at parietal electrodes (P3, Pz, P4). Except for incorrect penultimate stress in words with the structure 4 (V.(VC)(V:C); circled in Figure 5), the amplitude of positivity effects is significantly more pronounced in incorrect compared to correct conditions.

## Discussion

The current study aims at investigating whether speakers of Cairene Arabic are (like speakers of Turkish) partly insensitive to stress manipulations because stress in Cairene Arabic is predictable (as hypothesized in the Stress-“Deafness” account, i.e., Dupoux et al., 1997, 2001, 2008; Peperkamp and Dupoux, 2002), or whether the evaluation of stress differs between violations involving foot restructuring and those in which the prosodic structure is maintained.

In our ERP study utilizing a stress violation paradigm, violations of words with correct penultimate stress produced a positivity effect or a biphasic effect irrespective of prosodic structure: violations with antepenultimate stress evoked a positivity between 350 and 600 ms and violations with final stress a negativity between 400 and 550 ms and a positivity between 800 and 1150 ms. In contrast, for words with correct final stress asymmetrical results for different word structures are found: violations with antepenultimate stress evoked a positivity effect between 300 and 650 ms in both word types 3 and 4 and violations with penultimate stress a negativity between 400 and 480 ms, but a positivity only in word type 3 with the structure (V.V)(V:C) (between 550 and 850 ms).

We interpret the occurrence of positivity effects in different time-windows to reflect a task-related process that has been shown to reflect how easy it is for participants to decide how to classify a stress violation. We interpret these positivity effects as instances of the P3b family (Picton, 1992; Coulson et al., 1998; Niewenhuis et al., 2005) as found in previous similar experiments using the stress deviation paradigm (Domahs et al., 2008, 2013). The P3b effect is known to reflect stimulus probability, saliency, and task relevance in diverse cognitive domains. According to Coulson et al. (1998), the P300 is an appropriate dependent variable to test the saliency of a given manipulation because the amplitude and the latency of the effect increase with the degree of anomaly. Thus, in the present study, violations evoking enhanced positivity effects can be regarded as less probable than violations with reduced effects. Overall, the positivity effects observed vary in latency, most likely due to the fact that the evaluation and decision-making process is dependent on the perception of a stressed syllable. Since strong syllables play a crucial role in the perception of stress patterns, the latency differences can be explained by varying positions of stressed syllables.

Generally, the findings of the experiment reported in Section ERP Experiment on Cairene Arabic show that stress deviations in Cairene Arabic words produce brain responses reflecting the participants' sensitivity to most violations. Their brain responses are similar to those obtained in previous experiments on German and Turkish. In the following, the results for specific word structures will be discussed in comparison to previous results.

### Are speakers of Cairene Arabic insensitive to stress manipulations?

In Section *Previous ERP Studies on Word Stress Processing*, results reported for speakers of Turkish showed that Turkish participants had difficulties judging incorrect stress patterns if the default stress pattern was applied to words with lexical stress, while violations of words with canonical default stress produced enhanced positivity effects (Domahs et al., 2013). This finding was interpreted as evidence for the insensitivity to the default stress pattern, and for the view that the processing of stress information in Turkish mainly depends on the lexical status of stress (default vs. non-default stress). In Cairene Arabic, the position of word stress is also predictable though variable. In contrast to the Turkish default stress, stress in Cairene Arabic is not predictable by position but by structure. The behavioral data as well as the ERP data reported in Section *ERP Experiment on Cairene Arabic* suggest that speakers of Cairene Arabic are clearly sensitive to stress violations. In the behavioral data, correctly and incorrectly stressed words are accepted or rejected with an accuracy of more than 80%. Only violations involving incorrect antepenultimate stress are judged less accurately compared with other violations. However, this moderate difficulty is not reflected in ERPs in which violations with antepenultimate stress produced a positivity effect in each word type. In the study on Turkish, the condition with least accuracy in behavioral data did not produce a P300 effect.

In words with the structure 4 [V(VC)(V:C); e.g., ki.(rís)(ˈta:l)] with canonical final stress in Cairene Arabic, a lack of a positivity effect occurs for incorrect penultimate stress. We argue that the absence of a positivity cannot be explained by the factor predictability in the sense that penultimate stress is the default stress. In words like ki.(rís)(ˈta:l) final stress is the only predicted stress pattern. The most reasonable explanation is related to the metrical structure of phonological words in Cairene Arabic as discussed in the following section.

### The role of the metrical structure in stress processing

Related to the findings on German word stress processing (as summarized in Section *Previous ERP Studies on Word Stress Processing*), the second question was to test whether word stress processing in Cairene Arabic is guided by the internal foot structure of phonological words. In Table 5, the structures of correct forms are compared with those of incorrect forms.

**Table 5:** **Overview of metrical structures in correctly and incorrectly stressed forms and the occurrence of P3 effects as reflections of task-specific evaluation-to-expectation processes**.

**Canonical Structure**	**Structure violation**	**Restructuring of feet**	**Occurrence of P3**
1 V(ˈVC)V	*(ˈV)(VC)V	Yes	Yes
	*V(VC)(ˈV)	Yes	Yes
2 (VC)(ˈVC)V	*(ˈVC)(VC)V	No	Yes
	*(VC)(VC)(ˈV)	Yes	Yes
3 (V.V)(ˈVVC)	*(ˈV.V)(VVC)	No	Yes
	*V(ˈV)(VVC)	Yes	Yes
4 V(VC)(ˈVVC)	*(ˈV)(VC)(VVC)	Yes	Yes
	*V(ˈVC)(VVC)	No	No

In Table 5 it can be seen that in words exhibiting more than one foot (structures 2–4) violations occur that do not involve restructuring of feet, i.e., neither regrouping of syllables into feet nor creating feet from unparsed syllables. The results from the experiment on German (Domahs et al., 2008; see Section German) suggested a qualitative distinction between violations with stress realized on the head syllable of a weak foot and violations with stress on a weak or unparsed syllable. Thus, in German it was possible to identify indirectly which syllables are capable of bearing stress and which are not via the occurrence of P300 effects. With respect to the experiment on Cairene Arabic, it was expected that violations with stress on the head syllable of a weak foot are more difficult to classify as violation than violations involving changed structure, the latter ones leading to a P300 component. From the occurrences of P300 effects (Table 5) in the experiment on Cairene Arabic it seems that our hypothesis is not borne out in all cases: A lack of a P300 effect was obtained only for violations with penultimate stress when the structure was preserved (see final row in Table 5), but violations involving antepenultimate stress produce P300 effects in each word type, although in words with structure 2 and 3 such violations maintain the foot structure.

The question arises whether the effect patterns found in the study on Cairene Arabic can be interpreted along the same lines as the results found for German. We suggest that structure plays a role in Cairene Arabic stress processing when certain conditions are met: first the structure is maintained and second the incorrect stress pattern involved is a likely pattern in terms of frequency. Thus, we hypothesize that metrical structure is not the only factor influencing stress perception, but also the frequency asymmetries between different stress patterns. To strengthen this hypothesis we report the results of a frequency count on stress patterns in loan words.

An analysis of stress patterns in loan words in Cairene Arabic by El Shanawany (2013) showed that irrespective of the stress position in the source language, stress is assigned along the principles also suggested for native words of Cairene Arabic and is predictable by syllable quantity and position of (the head of) the final foot in phonological words. The corpus analyzed consisted of loan words because the trisyllabic stimuli presented in the ERP study are predominantly loans. Out of 286 types of bi-, tri-, and quadrisyllabic words, 57% exhibit final stress, 39% penultimate stress, and only 4% antepenultimate stress. Since native words of Cairene Arabic consist of higher proportions of mono- and bisyllabic words than loan words, the proportion of words with antepenultimate stress among native words can be expected to be even lower than 4%. Antepenultimate stress occurs only in words with three light syllables, a rare configuration. This corpus analysis demonstrates that final feet are more likely to be aligned with the right than with the left edge of phonological words. In this respect, Cairene Arabic differs from German for which it is postulated that the final foot within words is strong but which exhibits many exceptions with stress on non-final feet [e.g., 69% of existing words of the structure (V.V)(VC); see Janssen (2003)]. The positivity effect in words with incorrectly stressed head syllables in antepenultimate position (structures 2 and 3) indicate that such violations are clearly identified as deviating patterns though the participants were less accurate in explicitly judging them as incorrect compared to other violations. This discrepancy between behavioral and electrophysiological data suggests that the P300 effect not simply reflects the explicit judgment but rather the implicit evaluation of the likeliness of an event. One potential explanation for the occurrence of the P300 effect in words that preserve the prosodic structure could be that antepenultimate stress involving left aligned strong feet occur only rarely in Cairene Arabic and could therefore be classified as exceptional. In principle, the sensitivity to exceptional, less frequent stress patterns was also demonstrated in the study on Turkish word stress, in which only exceptional incorrect stress patterns led to P3 effects. Antepenultimate stress in Cairene Arabic is not exceptional in the sense that it is not derived by foot structure, but rather in terms of stress pattern frequency: only a few words consist of a sequence of three light syllables.

Taken together, the occurrence or absence of P3 effects in Cairene Arabic seems to be guided by the metrical structure and by the frequency distribution of the different stress positions, i.e., whether a certain pattern is exceptional or not. Therefore, we suggest that the participants' performance and sensitivity to word stress violations lie in between those observed for Turkish and German participants. Comparable to Turkish, exceptional stress patterns evoke a P3 effect when used incorrectly, and comparable to German, metrical structure plays a role. In contrast to Turkish, Cairene Arabic exhibits no default pattern, and in contrast to German word stress shows a stronger orientation toward the right edge of words.

### Negativity effect: error-detection mechanism or violation of lexical expectancy?

In Section Results, it was reported that violations involving penultimate and final stress evoked a biphasic ERP pattern. The discussion so far has mainly focused on the interpretation of the positivity effect. As regards the negativity effect in similar experiments, different interpretations have been proposed in the literature. In the study on German word stress processing (Domahs et al., 2008), an extended more fronto-centrally distributed negativity was found which was interpreted as an instance of a contingent negative variation (CNV; according to Rugg, 1984) to reflect the detection of a pitch-contour violation when a de-stressed initial syllable was encountered that did not provide sufficient information to judge such a form as incorrect. The judgment requires the detection of a stressed syllable (Domahs et al., 2008, 2013). In the present experiment on Cairene Arabic, however, the occurrences of negativity effects do not seem to mirror the perception of de-stressing and the prolonged activation of the phonological form in the working memory. The negativity effects occur for violations with penultimate and final stress, and in both cases the curve is not flat and extended over more than 400 ms (slow wave) but peaks at around 400–550 ms (see Figure 4).

In the study on Turkish word stress processing (Domahs et al., 2013), a centro-parietal negativity effect between 500 and 750 ms was obtained for violations with the default pattern (= final stress) replacing lexical penultimate stress. The effect was interpreted as belonging to the N400 family. For Turkish, it was assumed that exceptional stress on the penultimate or antepenultimate syllable has to be lexically specified in the phonological representations of words. If the lexical specification is not realized, the violation of the stress expectation leads to an N400 effect.

For Cairene Arabic, in contrast, it is not very likely that the negativity effects reflect deviations from lexical expectations. There are no indications that stress positions need to be lexically specified in Cairene Arabic. Furthermore, the components occur earlier than in the Turkish experiment (between 400 and 480 ms or 400–550 ms instead of 500–750 ms in Turkish). In previous studies on metrical processing (e.g., Koelsch et al., 2000; Rothermich et al., 2010), negativity effects were observed that have been proposed to indicate the general detection of deviations in metrical regularity or expectation. This component which has been described with different distributions (either lateralized or not, more frontally or broadly) and which has therefore been labeled differently, can be roughly summarized as an error detection component. It is suggested here that the present negativity effects represent an error detection mechanism, which is independent from lexical processing but related to metrical deviations. This component is independent from the occurrence of the later P3 effect as becomes evident for violations with penultimate stress in words with heavy penults [^*^V(ˈVC)(V:C) e.g., ^*^ki(rís)(ta:l)]. Thus, participants detect the metrical error, but in the evaluation process such violations are difficult to categorize as an unlikely form.

## Conclusion

The present behavioral and electrophysiological results on stress perception in Cairene Arabic show that speakers of this language are sensitive to stress information because they perform accurately in a stress evaluation task and produce ERP components indicating their ability to evaluate and categorize the likeliness of a certain stress pattern. Thus, psycholinguistic accounts of stress perception like the *Stress “Deafness*” account (i.e., Dupoux et al., 1997, 2001, 2008; Peperkamp and Dupoux, 2002), which assume that speakers of a language with predictable stress have difficulties identifying stress information, cannot explain the effect patterns we found.

Rather, our data support linguistic theories proposed for the Cairene Arabic word stress system as outlined in Section Metrical Properties of Cairene Arabic. In particular it was shown that prosodic structure, and metrical feet in particular, determines stress perception. This was evident for the processing of incorrect penultimate stress evoking a late positivity effect only if a light penult was stressed, but not when it was heavy. However, this structure effects cannot be generalized to incorrect antepenultimate stress which was easily categorized as unlikely irrespective of weight and its position within feet. To account for this result, it has been suggested that the frequency of stress patterns influences the processing of word stress in Cairene Arabic as a second factor. This hypothesis is supported by a corpus analysis of loan words. Effects of stress perception in Cairene Arabic lie therefore in between those obtained for German and Turkish.

Together with previous findings on stress perception in German and Turkish the present data complement the results by Dupoux, Peperkamp and colleagues that stress sensitivity is a function of predictability of stress. Our results suggest that the metrical structure in foot-based systems (i.e., German, Cairene Arabic), the lexical status of stress patterns in languages with default and lexical (exceptional) stress (i.e., Turkish), and the frequency of certain patterns also influences stress perception.

### Conflict of interest statement

The authors declare that the research was conducted in the absence of any commercial or financial relationships that could be construed as a potential conflict of interest.

## Figures and Tables

**Table A1:**
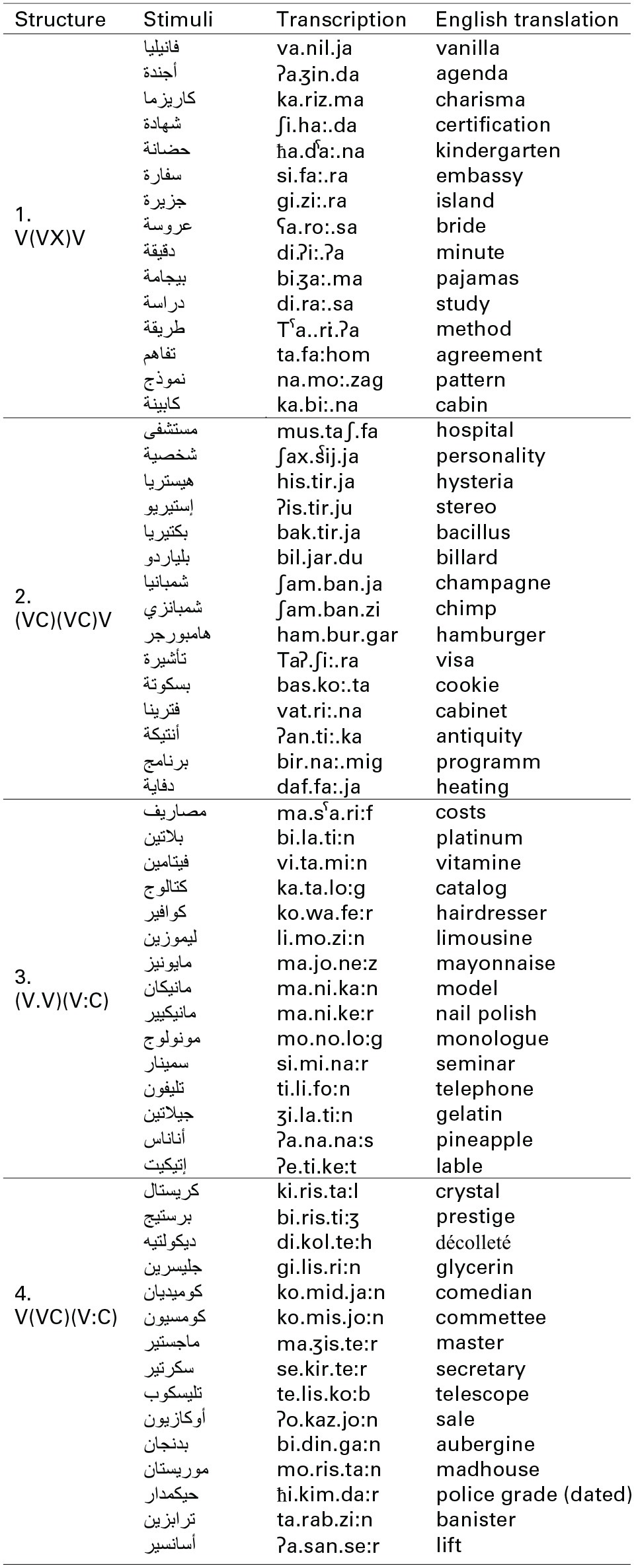
**List of critical items**.

## Appendix

### Appendix 2: overview over statistical results

Generalized repeated measures ANOVAs of mean voltage changes over the factors Foot Structure (the two structures per canonical stress pattern), Correctness (correct vs. incorrect stress condition) and Region (frontal, central, parietal electrodes) and *post-hoc* analyses of interactions with Bonferroni correction. Effect sizes are given in generalized eta-squared values (ges). Tables A2A–A2F provide summaries for separate comparisons of correct and incorrect conditions. U is the abbreviation for final stress, PU for penultimate stress and APU for antepenultimate stress. 1 and 2 refers to different foot structures.

**Table A2:** **Conditions and material**.

**(A) PU1/PU2 correct vs. incorrect APU stress/positivity in time-window 350 to 600 ms**					
**PU1: CV(CVC)CV**					
**PU2: (CVC)(CVC)CV**					
	**DFn**	**DFd**	***F***	***p***	**ges**
**Main effects**					
Region	2	38	6.273	**0.004**	0.033
Correctness	1	19	23.785	**0.000**	0.195
Foot structure	1	19	0.113	0.740	0.001
**Interactions**					
Region:Correctness	2	38	5.596	**0.007**	0.005
Region:Foot structure	2	38	0.332	0.720	0.000
Correctness:Foot structure	1	19	0.090	0.768	0.000
Region:Correctness:Foot structure	2	38	5.737	**0.007**	0.005
***Post-Hoc* Analyses (*p*-Values Bonferroni-Corrected)**					
Region:Correctness					
Frontal	1	19	28.27	**0.000**	0.286
Central	1	19	26.08	**0.000**	0.288
Parietal	1	19	13.02	**0.006**	0.288
Region:Correctness:Foot structure					
Foot Structure 1 frontal	1	19	39.04	**0.000**	0.327
Foot Structure 1 central	1	19	18.26	**0.001**	0.18
Foot Structure 1 parietal	1	19	8.003	**0.032**	0.074
Foot Structure 2 frontal	1	19	10.58	**0.013**	0.158
Foot Structure 2 central	1	19	23.01	**0.000**	0.33
Foot Structure 2 parietal	1	19	12.2	**0.007**	0.197
**(B) PU1/PU2 correct vs. incorrect U stress/negativity in time-window 400 to 550 ms**					
**PU1: CV(CVC)CV**					
**PU2: (CVC)(CVC)CV**					
	**DFn**	**DFd**	***F***	***p***	**ges**
**Main effect**					
Region	2	38	6.540	**0.004**	0.040
Correctness	1	19	8.998	**0.007**	0.083
Foot structure	1	19	0.053	0.821	0.000
**Interactions**					
Region:Correctness	2	38	2.947	0.065	0.003
Region:Foot structure	2	38	5.072	**0.011**	0.007
Correctness:Foot structure	1	19	3.895	0.063	0.018
Region:Correctness:Foot structure	2	38	0.482	0.621	0.000
***Post-Hoc* Analyses (*p*-Values Bonferroni-Corrected)**					
Region:Foot structure					
Frontal	1	19	2.066	0.501	0.023
Central	1	19	<1		0
Partietal	1	19	<1		0.006
**(C) PU1/PU2 correct vs. incorrect U stress/positivity in time-window 800 to 1150 ms**					
**PU1: CV(CVC)CV**					
**PU2: (CVC)(CVC)CV**					
	**DFn**	**DFd**	***F***	***p***	**ges**
**Main effects**					
Region	2	38	27.595	**0.000**	0.167
Correctness	1	19	9.265	**0.007**	0.043
Foot structure	1	19	0.169	0.686	0.001
**Interactions**					
Region:Correctness	2	38	35.409	**0.000**	0.031
Region:Foot structure	2	38	4.527	**0.017**	0.003
Correctness:Foot structure	1	19	3.057	0.097	0.012
Region:Correctness:Foot structure	2	38	7.326	**0.002**	0.004
***Post-Hoc* Analyses (*p*-Values Bonferroni-Corrected)**					
Region:Correctness:Word structure					
Foot Structure 1 frontal	1	19	3.47	0.234	0.05
Foot Structure 1 central	1	19	3.724	0.206	0.037
Foot Structure 1 parietal	1	19	15.4	**0.003**	0.136
Foot Structure 2 frontal	1	19	3.063	0.289	0.029
Foot Structure 2 central	1	19	12.3	**0.007**	0.092
Foot Structure 2 parietal	1	19	18.1	**0.001**	0.135
**(D) U1/U2 correct vs. incorrect APU stress/positivity in time-window 300 to 650 ms**					
**U1: (CV.CV)(CVVC)**					
**U2: CV(CVC)(CVVC)**					
	**DFn**	**DFd**	***F***	***p***	**ges**
**Main effects**					
Region	2	38	2.618	0.086	0.013
Correctness	1	19	70.228	**0.000**	0.270
Foot structure	1	19	0.955	0.341	0.004
**Interactions**					
Region:Correctness	2	38	7.630	**0.002**	0.007
Region:Foot structure	2	38	2.048	0.143	0.002
Correctness:Structure	1	19	4.480	**0.048**	0.012
Region:Correctnes:Foot structure	2	38	0.140	0.870	0.000
***Post-Hoc* Analyses (*p*-Values Bonferroni-Corrected)**					
Region:Correctness					
Frontal	1	19	59.07	**0.000**	0.359
Central	1	19	79	**0.000**	0.368
Parietal	1	19	45.26	**0.000**	0.205
Correctness:Foot Structure					
Foot Structure 1	1	19	74.58	**0.000**	0.465
Foot Structure 2	1	19	27.41	**0.000**	0.182
**(E) U1/U2 correct vs. incorrect APU stress/negativity in time-window 400 to 480 ms**					
**U1: (CV.CV)(CVVC)**					
**U2: CV(CVC)(CVVC)**					
**Main effects**	**DFn**	**DFd**	***F***	***p***	**ges**
Region	2	38	8.026	**0.001**	0.034
Correctness	1	19	6.957	**0.016**	0.060
Foot structure	1	19	5.002	**0.038**	0.013
**(E) U1/U2 correct vs. incorrect APU stress/negativity in time-window 400 to 480 ms**					
**U1: (CV.CV)(CVVC)**					
**U2: CV(CVC)(CVVC)**					
**Interactions**					
Region:Correctness	2	38	0.479	0.623	0.001
Region:Foot structure	2	38	0.048	0.953	0.000
Correctness:Foot structure	1	19	2.082	0.165	0.006
Region:Correctness:Foot structure	2	38	1.192	0.315	0.000
**(F) U1/U2 correct vs. incorrect PU stress/positivity in time-window 550 to 850 ms**					
**U1: (CV.CV)(CVVC)**					
**U2: CV(CVC)(CVVC)**					
	**DFn**	**DFd**	***F***	***p***	**ges**
**Main effects**					
Region	2	38	4.073	**0.025**	0.027
Correctness	1	19	4.552	**0.046**	0.041
Foot structure	1	19	0.094	0.763	0.000
**Interactions**					
Region:Correctness	2	38	4.250	**0.022**	0.005
Region:Foot structure	2	38	2.567	0.090	0.003
Correctness:Foot structure	1	19	7.909	**0.011**	0.011
Region:Correctness:Foot structure	2	38	2.749	0.077	0.001
***Post-Hoc* Analyses (*p*-Values Bonferroni-Corrected)**					
Region:Correctness					
Frontal	1	19	6.423	0.061	0.107
Central	1	19	4.325	0.154	0.05
Parietal	1	19	1.673	0.634	0.012
Correctness:Word structure					
Foot Structure 1	1	19	8.73	**0.016**	0.112
Foot Structure 2	1	19	1.031	0.645	0.012

## References

[B2] Coulson S. King J. W. Kutas M. (1998). Expect the unexpected: event-related brain responses to morphosyntactic violations. Lang. Cogn. Process. 13, 21–58. 10.1080/016909698386582

[B3] Domahs U. Genc S. Knaus J. Wiese R. Kabak B. (2013). Processing (un-)predictable word stress: ERP evidence from Turkish. Lang. Cogn. Process. 28, 335–354. 10.1080/01690965.2011.634590

[B4] Domahs U. Plag I. Carroll R. (2014). Word stress assignment in German, English and Dutch: quantity-sensitivity and extrametricality revisited. J. Comp. Ger. Linguist. 17, 59–96. 10.1007/s10828-014-9063-9

[B5] Domahs U. Wiese R. Bornkessel-Schlesewsky I. D. Schlesewsky M. (2008). The processing of German word stress: evidence for the prosodic hierarchy. Phonology 25, 1–36. 10.1017/S0952675708001383

[B6] Dupoux E. Pallier C. Sebastián N. Mehler J. (1997). A destressing “deafness” in French? J. Mem. Lang. 36, 406–421. 10.1006/jmla.1996.250017592731

[B7] Dupoux E. Peperkamp S. Sebastián-Gallés N. (2001). A robust method to study stress “deafness.” J. Acoust. Soc. Am. 110, 1606–1618. 10.1121/1.1380437 11572370

[B8] Dupoux E. Peperkamp S. Sebastián-Gallés N. (2010). Limits on bilingualism revisited: Stress ‘deafness’ simultaneous French-Spanish bilinguals. Cognition 114, 266–275. 10.1016/j.cognition.2009.10.001 19896647

[B9] Dupoux E. Sebastián-Gallés N. Navarrete E. Peperkamp S. (2008). Persistent stress ‘deafness’: the case of French learners of Spanish. Cognition 106, 682–706. 10.1016/j.cognition.2007.04.001 17592731

[B10] Eisenberg P. (1991). Syllabische Struktur und Wortakzent: Prinzipien der Prosodik deutscher Wörter. Z. Sprachwissenschaft 10, 37–64. 10.1515/zfsw.1991.10.1.37

[B11] El Shanawany H. (2013). Der Kairo-Arabische Wortakzent im Vergleich Zum Deutschen: Eine EEG-Untersuchung [A Comparison Between Cairene-Arabic and German Word Stress: an ERP Study]. Ph.D. dissertation, Philipps-Universität Marburg. Available online at: http://archiv.ub.uni-marburg.de/diss/z2014/0068/

[B12] Féry C. (1998). German word stress in optimality theory. J. Comp. Ger. Linguist. 2. 101–142. 10.1023/A:1009883701003

[B13] Göksel A. Kerslake C. (2005). Turkish: A Comprehensive Grammar. London/New York: Routledge

[B15] Harrell R. (1960). A linguistic analysis of egyptian radio arabic, in Contributions to Arabic Linguistics, ed Ferguson C. A. (Cambridge, MA: Harvard University Press), 3–77

[B16] Hayes B. (1995). Metrical Stress Theory: Principles and Case Studies. Chicago: University of Chicago Press

[B17] Hulst van der H. Hellmuth S. (2010). Word accent systems in the middle east, in A Survey of Word Accentual Patterns in the Languages of the World, chap. 11, eds Hulst van der H. Goedemans R. van Zanten E. (Berlin; New York: Mouton de Gruyter), 615–646

[B18] Hyman L. (1985). A Theory of Phonological Weight. Dordrecht: Foris

[B19] Inkelas S. (1999). Exceptional stress-attracting suffixes in Turkish: representations versus the grammar, in The Prosody-Morphology Interface, eds Kager R. Hulst van der H. Zonneveld W. (Cambridge: Cambridge University Press), 134–187

[B20] Inkelas S. Orgun C. O. (2003). Turkish stress: a review. Phonology 20, 139–161. 10.1017/S0952675703004482

[B21] Janssen U. (2003). Untersuchungen Zum Wortakzent im Deutschen und Niederländischen. Düsseldorf: Heinrich-Heine-Universität Düsseldorf

[B22] Kabak B. Vogel I. (2001). The phonological word and stress assignment in Turkish. Phonology 18, 315–360. 10.1017/S0952675701004201

[B23] Kabak B. Vogel I. (2011). Exceptions to stress and harmony: cophonologies or prespecification? in Expecting the Unexpected: Exceptions in Grammar, eds Simon H. J. Wiese H. (Berlin/New York: Mouton de Gruyter), 59–94

[B23a] Kaisse E. (1985). Some theoretical consequences of stress rules in Turkish, in Papers from the General Session of the 21st Regional Meeting, eds Eilfort W. Kroeber P. Peterson K. (Chicago, IL: Chicago Linguistic Society), 199–209

[B24] Knaus J. Domahs U. (2009). Experimental evidence for optimal and minimal metrical structure of German word prosody. Lingua 119, 1396–1413. 10.1016/j.lingua.2008.04.002

[B25] Knaus J. Wiese R. Janssen U. (2007). The processing of word stress: EEG studies on task-related components, in Proceedings of the 16th International Conference of Phonetic Sciences (Saarbrücken), 709–712

[B26] Koelsch S. Gunter T. Friederici A. D. Schröger E. (2000). Brain indices of music processing: “Nonmusicians” are musical. J. Cogn. Neurosci. 12, 520–541. 10.1162/089892900562183 10931776

[B27] Kornfilt J. (1997). Turkish. London; New York: Routledge

[B28] Kutas M. Federmeier K. D. (2011). Thirty years and counting: finding meaning of the N400 component of the event-related brain potential (ERP). Annu. Rev. Psychol. 62, 621–647. 10.1146/annurev.psych.093008.131123 20809790 PMC4052444

[B30a] Lewis G. (1967/2000). Turkish Grammar. Oxford: Oxford University Press

[B32] Marie C. Magne C. Besson M. (2011). Musicians and the metric structure of words. J. Cogn. Neurosci. 23, 294–305. 10.1162/jocn.2010.21413 20044890

[B33] McCarthy J. (1979). Formal Problems in Semitic Phonology and Morphology. Ph.D. dissertation, MIT.

[B34] Mitchell T. F. (1960). Prominence and syllabification in Arabic. Bull. Sch.Orient. Afr. Stud. 2, 369–389. 10.1017/S0041977X00149997

[B35] Niewenhuis S. Aston-Jones G. Cohen J. D. (2005). Decision making, the P3, and the locus coeruleus–norepinephrine system. Psychol. Bull. 131, 510–532. 10.1037/0033-2909.131.4.510 16060800

[B36] Peperkamp S. Dupoux E. (2002). A typological study of stress ‘deafness’, in Laboratory Phonology 7, eds Gussenhoven C. Warner N. (Berlin: Mouton de Gruyter), 203–240

[B37] Peperkamp S. Vendelin I. Dupoux E. (2010). Perception of predictable stress: a cross-linguistic investigation. J. Phon. 38, 422–430. 10.1016/j.wocn.2010.04.001

[B38] Picton T. W. (1992). The P300 wave of the human event-related brain potential. J. Clin. Neurophysiol. 9, 456–479. 10.1097/00004691-199210000-00002 1464675

[B40] Rothermich K. Schmidt-Kassow M. Schwartze M. Kotz S. A. (2010). Event-related potential responses to metric violations: rules versus meaning. Neuroreport 21, 580–584. 10.1097/WNR.0b013e32833a7da7 20431495

[B39] Röttger T. B. Domahs U. Grande M. Domahs F. (2012). Structural factors affecting the assignment of word stress in German. J. Ger. Linguist. 24, 53–94. 10.1017/S1470542711000262

[B41] Rugg M. D. (1984). Event-related potentials in phonological matching tasks. Brain Lang. 23, 225–240. 10.1016/0093-934X(84)90065-8 6518354

[B42] Rugg M. D. Nagy M. E. (1989). Event-related potentials and recognition memory for words Electroencephalogr. Clin. Neurophysiol. 72, 395–406. 2469564 10.1016/0013-4694(89)90045-x

[B42a] Schmidt-Kassow M. Roncaglia-Denissen M. P. Kotz S. (2011a). Why pitch sensitivity matters: event-related potential evidence of metrical and syntactic violation detection among Spanish late learners of German. Front. Psychol. 2:131. 10.3389/fpsyg.2011.00131 21734898 PMC3120976

[B42b] Schmidt-Kassow M. Rothermich K. Schwartze M. Kotz S. (2011b). Did you get the beat? Late proficient French-German learners extract strong-weak patterns in tonal but not in linguistic sequences. Neuroimage 54, 568–576. 10.1016/j.neuroimage.2010.07.062 20692349

[B43] Sezer E. (1981). On non-final stress in Turkish. J. Turkish Stud. 5, 61–69

[B45] Watson J. C. E. (2002). The Phonology and Morphology of Arabic. Oxford: Oxford University Press

[B46] Wiese R. (1996). The Phonology of German. Oxford: Oxford University Press

[B47] Woidich M. (2006). Das Kairenisch-Arabische. Eine Grammatik. Wiesbaden: Harrassowitz Verlag

